# A “Maioridade” do Escore de Cálcio Coronário?

**DOI:** 10.36660/abc.20230708

**Published:** 2023-11-14

**Authors:** António Miguel Ferreira, Rita Lima, Nuno Bettencourt

**Affiliations:** 1 Hospital de Santa Cruz Lisboa Portugal Hospital de Santa Cruz, Carnaxide, Lisboa – Portugal; 2 Hospital da Luz Lisboa Portugal Hospital da Luz, Lisboa – Portugal; 3 Universidade do Porto Faculdade de Medicina Unidade de Investigação Cardiovascular Porto Portugal Universidade do Porto Faculdade de Medicina – Unidade de Investigação Cardiovascular, Porto – Portugal

**Keywords:** Fatores Etários, Aterosclerose, Cálcio

***“Um homem é tão velho quanto suas artérias”***

***Thomas Sydenham (médico inglês, 1624-1689)***

O uso do escore de cálcio arterial coronariano (CAC) para orientar a prevenção primária da aterosclerose e suas manifestações tem crescido em popularidade nos últimos anos. Suas vantagens sobre o paradigma atual de calculadoras baseadas em fatores de risco incluem melhor discriminação e maior poder preditivo, com a promessa de melhores decisões de tratamento e implementação oportuna de medidas preventivas personalizadas.^1-3^

Os resultados da CAC são geralmente expressos em valor absoluto e em percentil entre indivíduos da mesma idade e sexo. É importante ressaltar que transmitir essas informações aos pacientes parece melhorar sua adesão às modificações no estilo de vida e à terapia farmacológica.^4^ Uma das formas desenvolvidas para fazer os pacientes compreenderem seu risco é o conceito de idade vascular. Muito simplesmente, a idade vascular de um indivíduo avaliada pelo escore CAC é a idade média em que pessoas do mesmo sexo apresentam grau semelhante de calcificação coronariana. Portanto, se a idade vascular de uma pessoa for superior à idade cronológica, isso indica um risco maior de eventos cardiovasculares do que se poderia supor. Por exemplo, uma mulher fumadora de 50 anos com uma idade vascular de 70 anos pode perceber que precisa tomar medidas preventivas para reduzir o seu risco. Por outro lado, quando a idade vascular é inferior à idade cronológica, sugere um risco menor em comparação com a sua faixa etária.^5^ Embora a idade vascular forneça uma forma clara e identificável de comunicar o risco cardiovascular aos pacientes, deve notar-se que pode transmitir a falsa noção que a aterosclerose faz parte do processo natural de envelhecimento. Não faz. No entanto, apesar desta imprecisão, a sua utilidade clínica permanece.

Nesta edição dos ABC, um interessante estudo procurou avaliar o impacto do uso da idade vascular para reclassificar o risco de 150 homens brancos assintomáticos submetidos a escores de CAC.^6^ A substituição da idade cronológica pela idade vascular na equação de coorte agrupada estado-unidense resultou na reclassificação de dois terços dos sujeitos (31% para cima e 36% para baixo). Na ausência de eventos clínicos para avaliar a adequação desta reclassificação, os autores utilizaram a progressão do escore CAC em um segundo exame (realizado quase 8 anos depois, em média) como substituto. Ao contrário dos escores de risco basais, a idade vascular foi significativamente correlacionada com a progressão da CAC ao longo do tempo.

Este estudo ilustra uma das aplicações potenciais da idade vascular (ou seja, para ser usada em vez da idade cronológica no cálculo dos escores de risco). Além disso, apoia a noção de que o escore CAC supera as calculadoras atuais apoiadas pelas diretrizes na avaliação do risco cardiovascular. No entanto, várias limitações também devem ser reconhecidas. Usar um parâmetro baseado em CAC, como a idade vascular, para prever a progressão do CAC é, até certo ponto, uma promessa autorrealizável, uma vez que o CAC basal é provavelmente o melhor preditor da progressão do CAC ao longo do tempo.^7^ Outra advertência importante é a ausência de dados sobre medicamentos hipolipemiantes no início do estudo e durante o período entre exames. Sabe-se que as estatinas aumentam os escores de CAC em alguns pacientes através de um mecanismo que provavelmente envolve a “estabilização” de placas pré-existentes com calcificação concomitante.^8^ Portanto, a progressão do CAC pode ser desejável em certas circunstâncias, o que é uma das razões pelas quais a interpretação de pós- exames repetidos de estatina são problemáticos.

Apesar dessas limitações, este estudo nos lembra dos benefícios potenciais da avaliação da aterosclerose subclínica com o escore CAC e das possíveis formas de integrar essas informações à nossa prática clínica atual de estimativa de risco com calculadoras. Vários grandes ensaios randomizados em andamento nos dirão em breve se devemos continuar usando CAC apenas em casos selecionados (quando o benefício da terapia farmacológica é incerto) ou se esta nova abordagem deve se tornar padrão. Fique atento!

## References

[1] Wong ND (2022). Evolution of Coronary Calcium Screening for Assessment of Atherosclerotic Cardiovascular Disease Risk and Role in Preventive Cardiology. Curr Atheroscler Rep.

[2] Bettencourt N, Mendes L, Fontes JP, Matos P, Ferreira C, Botelho A (2022). Consensus Document on Chronic Coronary Syndrome Assessment and Risk Stratification in Portugal: A Position Paper Statement from the [Portuguese Society of Cardiology’s] Working Groups on Nuclear Cardiology, Magnetic Resonance and Cardiac Computed Tomography, Echocardiography, and Exercise Physiology and Cardiac Rehabilitation. Rev Port Cardiol.

[3] Valério RS, Generoso G, Fernandes JL, Nasir K, Hong JC, Bittencourt MS (2022). Cost-Effectiveness of Using the Coronary Artery Calcium Score in Guiding Therapeutic Decisions in Primary Prevention in the Brazilian Population. Arq Bras Cardiol.

[4] Gupta A, Lau E, Varshney R, Hulten EA, Cheezum M, Bittencourt MS (2017). The Identification of Calcified Coronary Plaque Is Associated with Initiation and Continuation of Pharmacological and Lifestyle Preventive Therapies: A Systematic Review and Meta-Analysis. JACC Cardiovasc Imaging.

[5] McClelland RL, Nasir K, Budoff M, Blumenthal RS, Kronmal RA (2009). Arterial Age as a Function of Coronary Artery Calcium (from the Multi-Ethnic Study of Atherosclerosis [MESA]). Am J Cardiol.

[6] Polli I, Bruscato NM, Da Luz PL, Freitas DDM, Almeida AO, Carli W (2023). Determination of Vascular Age in Men Using the Coronary Calcium Score and its Impact on Restratification of Cardiovascular Risk. Arq Bras Cardiol.

[7] Erbel R, Lehmann N, Churzidse S, Rauwolf M, Mahabadi AA, Möhlenkamp S (2014). Progression of Coronary Artery Calcification Seems to be Inevitable, but Predictable - Results of the Heinz Nixdorf Recall (HNR) study. Eur Heart J.

[8] Puri R, Nicholls SJ, Shao M, Kataoka Y, Uno K, Kapadia SR (2015). Impact of Statins on Serial Coronary Calcification During Atheroma Progression and Regression. J Am Coll Cardiol.

