# Supplementary material for: A “Maioridade” do Escore de Cálcio Coronário?
Source: Arq Bras Cardiol. 2023 Nov 14;120(10):e20230708. [Article in Portuguese] doi: 10.36660/abc.20230708 (PMC10697674; doi:10.36660/abc.20230708)
Supplement: Translation

# Translation: The “Coming of Age” of Coronary Calcium Score?

**Authors:** António Miguel Ferreira, Rita Lima, Nuno Bettencourt

**Affiliations:** 1 Portugal Hospital de Santa Cruz, Carnaxide, Lisboa – Portugal; 2 Portugal Hospital da Luz, Lisboa – Portugal; 3 Portugal Universidade do Porto Faculdade de Medicina – Unidade de Investigação Cardiovascular, Porto – Portugal

**Keywords:** Age Factors, Atherosclerosis, Calcium

***“A man is as old as his arteries” ***

***Thomas Sydenham (English physician, 1624-1689)***

The use of coronary artery calcium (CAC) score to guide the primary prevention of atherosclerosis and its manifestations has grown in popularity in recent years. Its advantages over the current paradigm of risk-factor-based calculators include better discrimination and greater predictive power, with the promise of better treatment decisions and timely implementation of personalized preventive measures.^1-3^

CAC results are usually expressed as an absolute value and as a percentile among individuals of the same age and sex. Importantly, conveying this information to patients seems to improve their adherence to lifestyle modifications and pharmacological therapy.^4^ One of the ways developed for making patients understand their risk is the concept of vascular age. Quite simply, an individual’s vascular age assessed by CAC score is the average age at which people of the same sex have a similar degree of coronary calcification. So, if a person’s vascular age is higher than their chronological age, this indicates a higher risk of cardiovascular events than one would suppose. For instance, a 50-year-old female smoker with a vascular age of 70 may realize that she needs to take preventive measures to reduce her risk. Conversely, when vascular age is lower than chronological age, it suggests a lower risk compared to their age group.^5^ Although vascular age provides a clear and relatable way to communicate cardiovascular risk to patients, it should be noted that it might convey the false notion that atherosclerosis is part of the natural aging process. It is not. Nevertheless, despite this imprecision, its clinical usefulness remains.

In this issue of ABC, an interesting study sought to assess the impact of using vascular age to reclassify the risk of 150 asymptomatic white men undergoing CAC scores.^6^ Replacing chronological age with vascular age in the North American Pooled Cohort Equation resulted in the reclassification of two-thirds of the subjects (31% upward and 36% downward). In the absence of clinical events to assess the appropriateness of this reclassification, the authors used CAC score progression on a second scan (performed almost 8 years later, on average) as a surrogate. Unlike baseline risk scores, vascular age was significantly correlated with CAC progression over time.

This study illustrates one of the potential applications of vascular age (i.e., to be used instead of chronological age when calculating risk scores). Also, it supports the notion that the CAC score outperforms current guideline-supported calculators in assessing cardiovascular risk. However, several limitations should also be acknowledged. Using a CAC-based parameter such as vascular age to predict CAC progression is, to some degree, a self-fulfilling promise since baseline CAC is probably the best predictor of CAC progression over time.^7^ Another important caveat is the absence of data on lipid-lowering medication at baseline and during the inter-scan period. Statins are known to increase CAC scores in some patients through a mechanism that probably involves “stabilization” of pre-existing plaques with concurrent calcification.^8^ So, CAC progression may be desirable in certain circumstances, which is one reason why the interpretation of post-statin repeat scans is troublesome.

Despite these limitations, this study reminds us of the potential benefits of assessing subclinical atherosclerosis with CAC score and possible ways of integrating this information with our current clinical practice of estimating risk with calculators. Several ongoing large randomized trials will soon tell us whether we should keep using CAC only in selected cases (when the benefit of pharmacological therapy is uncertain) or if this new approach should become standard. Stay tuned!
